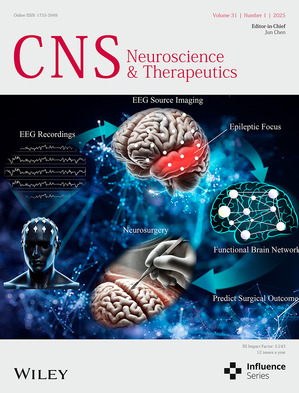# Front Cover

**DOI:** 10.1111/cns.70254

**Published:** 2025-02-04

## Abstract

Cover image: The cover image is based on the article *Source Causal Connectivity Noninvasively Predicting Surgical Outcomes of Drug‐Refractory Epilepsy* by Yalin Wang et al., https://doi.org/10.1111/cns.70196.